# A multiresolution approach to automated classification of protein subcellular location images

**DOI:** 10.1186/1471-2105-8-210

**Published:** 2007-06-19

**Authors:** Amina Chebira, Yann Barbotin, Charles Jackson, Thomas Merryman, Gowri Srinivasa, Robert F Murphy, Jelena Kovačević

**Affiliations:** 1 Center for Bioimage Informatics and Dept. of Biomedical Engineering, Carnegie Mellon University, Pittsburgh, PA, USA; 2 Dept. of Electrical and Computer Engineering, Carnegie Mellon University, Pittsburgh, PA, USA; 3 Depts. of Biological Sciences and Machine Learning, Carnegie Mellon University, Pittsburgh, PA, USA; 4 Dept. of Communication Systems, Swiss Federal Institute of Technology, Lausanne, Switzerland

## Abstract

**Background:**

Fluorescence microscopy is widely used to determine the subcellular location of proteins. Efforts to determine location on a proteome-wide basis create a need for automated methods to analyze the resulting images. Over the past ten years, the feasibility of using machine learning methods to recognize all major subcellular location patterns has been convincingly demonstrated, using diverse feature sets and classifiers. On a well-studied data set of 2D HeLa single-cell images, the best performance to date, 91.5%, was obtained by including a set of multiresolution features. This demonstrates the value of multiresolution approaches to this important problem.

**Results:**

We report here a novel approach for the classification of subcellular location patterns by classifying in multiresolution subspaces. Our system is able to work with any feature set and any classifier. It consists of multiresolution (MR) decomposition, followed by feature computation and classification in each MR subspace, yielding local decisions that are then combined into a global decision. With 26 texture features alone and a neural network classifier, we obtained an increase in accuracy on the 2D HeLa data set to 95.3%.

**Conclusion:**

We demonstrate that the space-frequency localized information in the multiresolution subspaces adds significantly to the discriminative power of the system. Moreover, we show that a vastly reduced set of features is sufficient, consisting of our novel modified Haralick texture features. Our proposed system is general, allowing for any combinations of sets of features and any combination of classifiers.

## Background

### Automated interpretation of protein subcellular location

Among the most important goals in biological sciences today is to understand the function of all proteins. One of the critical characteristics of a protein is its subcellular location, that is, its spatial distribution within the cell. Knowledge of the location of all proteins will be essential for building accurate models that capture and simulate cell behavior, and eventually can be expected to be useful for early diagnosis of disease and/or monitoring of therapeutic effectiveness. The most widely used method for determining protein subcellular location is fluorescence microscopy. Given that mammalian cells are believed to express tens of thousands of proteins, comprehensive analysis of protein location will require acquisition of numbers of images that are beyond our ability to analyze visually.

Fortunately, the feasibility of automated interpretation of subcellular patterns in fluorescence microscope images has been clearly demonstrated over the past ten years, initially by our group [1-3] and then by others [4-6]. The result is systems that can classify protein location patterns with well-characterized reliability and better sensitivity than human observers (for reviews, please see [7,8]). The heart of such systems is a set of numerical features – Subcellular Location Features (SLFs) – to describe the spatial distribution of proteins in each cell image. The SLFs include Haralick texture features, morphological features, and Zernike moments. Of particular relevance to the work described here is that the addition of simple multiresolution features resulted in a significant improvement of classification accuracy, to the highest reported accuracy of 91.5% for the 2D HeLa data set. This dataset contains images of all major subcellular patterns and is a well-established testbed for evaluating subcellular pattern analysis approaches. Note that with the aid of a parallel DNA channel, that accuracy climbed to 92.0%. It is important to have methods that work well when DNA images are available and also when they are not. We focus here on analysis of patterns without parallel DNA images and on improving performance relative to the best previous results.

### Multiresolution techniques

As the introduction of the simplest *multiresolution (MR) *features produced a statistically significant jump in classification accuracy, our aim is to explore more sophisticated multiresolution techniques. In particular, the following are the three characteristics of multiresolution [9,10] we wish to explore: 

(a) Localization: Fluorescence microscope images have highly localized structures both in space and frequency. This leads us to MR tools, as they have been found to be the most appropriate tools for computing and isolating such localized structures [11].

(b) Adaptivity: Given that we are designing a system to distinguish between classes of proteins, it is clear that an ideal solution is to use adaptive transforms, a property provided by MR techniques. The reasoning is that if there is a different MR transform for each different class, then the transform itself would help in distinguishing the class.

(c) Fast and Efficient Computation: It is well known that MR techniques such as wavelets have a computational cost of the order *O*(*N*), where *N *is the input size, as opposed to *O*(*N *log *N*) typical for other linear transforms including the FFT. This is one of the major reasons for the phenomenal success of MR techniques in real applications and one of the reasons to incorporate MR features into the system. 

MR transforms are many; we now give a brief overview. The basic idea behind MR is that we can look at a signal at different scales or resolutions and at different points in time. This should give us information about hidden structures in the signal, with a particular behavior across scales.

**Figure 1:**
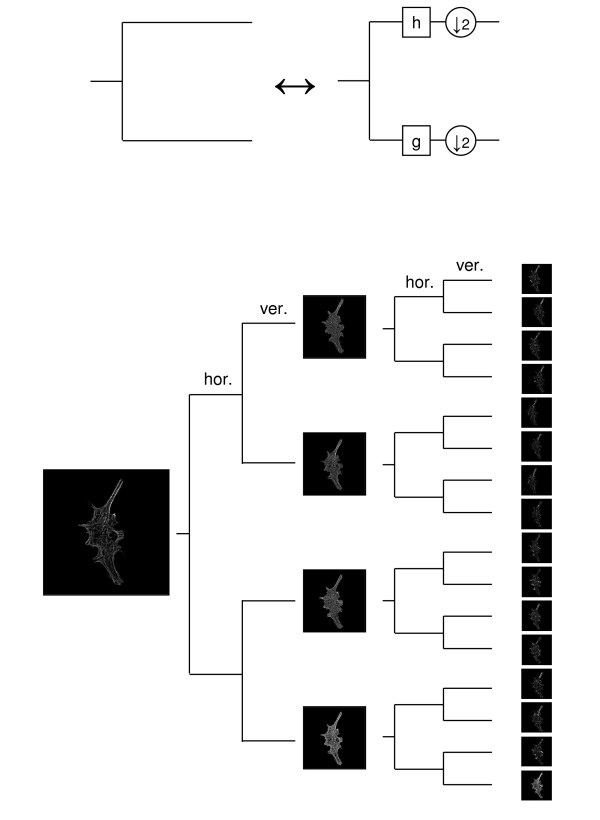
**Basic multiresolution block**. Top: Two-channel analysis filter bank. The filter *h *is a highpass filter and *g *is a lowpass filter. Bottom: A 2-level filter bank decomposition of actin. If the original image is of size *N *× *N*, the ones in the middle are of sizes *N*/2 × *N*/2 and the ones on the right are of sizes *N*/4 × *N*/4. Each branch has either the lowpass filter *g *or the highpass filter *h *followed by downsampling by 2 as in the top figure. Filtering and sampling are performed along the horizontal direction (rows) followed by the same operations along the vertical direction (columns).

The main building block of any MR transform is a *filter *bank [10]; it is a device that splits the signal into *MR subspaces *(also called subbands, wavelet coefficients or transform coefficients), where each MR subspace contains one part of the signal's spectrum. As an example, in the top part of Figure 1, a two-channel filter bank is given, operating on a 1D signal. For images, on both rows and columns (horizontal and vertical directions), the signal is filtered, followed by downsampling by two (discarding every other sample, allowed because there is filtering beforehand). In the simplest case, this produces four subbands; one extracting lowpass information in both directions, one extracting highpass information in both direction and the remaining two extracting lowpass information in one direction and the highpass information in the other. The example in the lower part of the figure shows such a filter bank applied to each subband again (two levels), as we will use in this paper.

Adaptivity of MR transforms manifests itself in many guises, including a number of popular transforms: (a) Growing a full tree to *L *levels with specific filters of the same length as the downsampling factor yields the Discrete Fourier Transform (DFT) of size 2^*L*^. (b) Growing a full tree to *L *levels but allowing the filters to be longer, leads to the Short-Time Fourier Transform, or, the Gabor Transform. (c) Growing the tree only on the lowpass branch to *L *levels leads to the *L*-level Discrete Wavelet Transform (DWT). (d) Growing an arbitrary tree leads to Wavelet Packets (WP). (e) Splitting the signal into more than two channels, allowing filters in the above transforms to be orthogonal and/or linear phase, allowing for true multidimensional filters and/or samplers, etc., leads to even more degrees of freedom.

### Towards multiresolution classification

**Figure 2:**
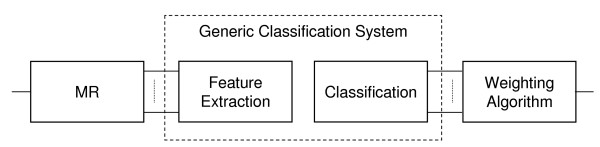
**Multiresolution (MR) classification system**. The generic classification system (GCS) consists of feature extraction followed by classification (inside the dashed box). We add an MR block in front of GCS and compute features in MR subspaces (subbands). Classification is then performed on each of the subbands yielding local decisions which are then weighed and combined to give a final decision.

We now summarize our initial MR classification effort [12,13]. We started with a simple classification system consisting of Haralick texture feature computation followed by a maximum-likelihood classifier, and demonstrated that, by adding an MR block in front, we were able to raise the classification accuracy by roughly 10% (from 71.8% to 82.2%) as compared to the system with no MR. This fits within our generic framework shown in Figure 2, where the feature computation block uses Haralick texture features and the classification block is maximum likelihood. We concluded that selecting features in MR subspaces allows us to custom-build discriminative feature sets. However, although the multiresolution block substantially increased classification accuracy, the accuracy of the overall system was still not high enough, and thus, in this work, we reexamined each step of the system: the features used, the classifier, and the weighting process.

## Results and discussion

### Problem statement and philosophy

The problem we are addressing is that of classifying the spatial distribution patterns of selected proteins within the cell. Assume that the images are of size *N *× *N *and let ℝ denote the set of intensities covered by all the images in the given dataset, compactly represented as an image belonging to ℝ^*N *× *N*^. Then, the problem can be formulated as designing a map from the *signal space *of protein localization images X
 MathType@MTEF@5@5@+=feaafiart1ev1aaatCvAUfKttLearuWrP9MDH5MBPbIqV92AaeXatLxBI9gBamrtHrhAL1wy0L2yHvtyaeHbnfgDOvwBHrxAJfwnaebbnrfifHhDYfgasaacH8akY=wiFfYdH8Gipec8Eeeu0xXdbba9frFj0=OqFfea0dXdd9vqai=hGuQ8kuc9pgc9s8qqaq=dirpe0xb9q8qiLsFr0=vr0=vr0dc8meaabaqaciaacaGaaeqabaWaaeGaeaaakeaaimaacqWFxepwaaa@384F@ ⊂ ℝ^*N *× *N*^, to a *response space *Y
 MathType@MTEF@5@5@+=feaafiart1ev1aaatCvAUfKttLearuWrP9MDH5MBPbIqV92AaeXatLxBI9gBamrtHrhAL1wy0L2yHvtyaeHbnfgDOvwBHrxAJfwnaebbnrfifHhDYfgasaacH8akY=wiFfYdH8Gipec8Eeeu0xXdbba9frFj0=OqFfea0dXdd9vqai=hGuQ8kuc9pgc9s8qqaq=dirpe0xb9q8qiLsFr0=vr0=vr0dc8meaabaqaciaacaGaaeqabaWaaeGaeaaakeaaimaacqWFyeFwaaa@3851@⊆{1, 2,..., *C*} of class labels. Thus, decision *d *is the map, *d*: X
 MathType@MTEF@5@5@+=feaafiart1ev1aaatCvAUfKttLearuWrP9MDH5MBPbIqV92AaeXatLxBI9gBamrtHrhAL1wy0L2yHvtyaeHbnfgDOvwBHrxAJfwnaebbnrfifHhDYfgasaacH8akY=wiFfYdH8Gipec8Eeeu0xXdbba9frFj0=OqFfea0dXdd9vqai=hGuQ8kuc9pgc9s8qqaq=dirpe0xb9q8qiLsFr0=vr0=vr0dc8meaabaqaciaacaGaaeqabaWaaeGaeaaakeaaimaacqWFxepwaaa@384F@ ↦ Y
 MathType@MTEF@5@5@+=feaafiart1ev1aaatCvAUfKttLearuWrP9MDH5MBPbIqV92AaeXatLxBI9gBamrtHrhAL1wy0L2yHvtyaeHbnfgDOvwBHrxAJfwnaebbnrfifHhDYfgasaacH8akY=wiFfYdH8Gipec8Eeeu0xXdbba9frFj0=OqFfea0dXdd9vqai=hGuQ8kuc9pgc9s8qqaq=dirpe0xb9q8qiLsFr0=vr0=vr0dc8meaabaqaciaacaGaaeqabaWaaeGaeaaakeaaimaacqWFyeFwaaa@3851@ that associates an input image with a class label [14]. To reduce the dimensionality of the problem, one sets up a feature space ℱ
 MathType@MTEF@5@5@+=feaafiart1ev1aaatCvAUfKttLearuWrP9MDH5MBPbIqV92AaeXatLxBI9gBamrtHrhAL1wy0L2yHvtyaeHbnfgDOvwBHrxAJfwnaebbnrfifHhDYfgasaacH8akY=wiFfYdH8Gipec8Eeeu0xXdbba9frFj0=OqFfea0dXdd9vqai=hGuQ8kuc9pgc9s8qqaq=dirpe0xb9q8qiLsFr0=vr0=vr0dc8meaabaqaciaacaGaaeqabaWaaeGaeaaakeaaimaacqWFXeIraaa@3787@⊂ ℝ^*f*^, *f *≤ *N*^2^, between the input space and the response space. The feature extractor *θ *is the map *θ*:X
 MathType@MTEF@5@5@+=feaafiart1ev1aaatCvAUfKttLearuWrP9MDH5MBPbIqV92AaeXatLxBI9gBamrtHrhAL1wy0L2yHvtyaeHbnfgDOvwBHrxAJfwnaebbnrfifHhDYfgasaacH8akY=wiFfYdH8Gipec8Eeeu0xXdbba9frFj0=OqFfea0dXdd9vqai=hGuQ8kuc9pgc9s8qqaq=dirpe0xb9q8qiLsFr0=vr0=vr0dc8meaabaqaciaacaGaaeqabaWaaeGaeaaakeaaimaacqWFxepwaaa@384F@ ↦ ℱ
 MathType@MTEF@5@5@+=feaafiart1ev1aaatCvAUfKttLearuWrP9MDH5MBPbIqV92AaeXatLxBI9gBamrtHrhAL1wy0L2yHvtyaeHbnfgDOvwBHrxAJfwnaebbnrfifHhDYfgasaacH8akY=wiFfYdH8Gipec8Eeeu0xXdbba9frFj0=OqFfea0dXdd9vqai=hGuQ8kuc9pgc9s8qqaq=dirpe0xb9q8qiLsFr0=vr0=vr0dc8meaabaqaciaacaGaaeqabaWaaeGaeaaakeaaimaacqWFXeIraaa@3787@, and the classifier *ψ *is the map *ψ*: ℱ
 MathType@MTEF@5@5@+=feaafiart1ev1aaatCvAUfKttLearuWrP9MDH5MBPbIqV92AaeXatLxBI9gBamrtHrhAL1wy0L2yHvtyaeHbnfgDOvwBHrxAJfwnaebbnrfifHhDYfgasaacH8akY=wiFfYdH8Gipec8Eeeu0xXdbba9frFj0=OqFfea0dXdd9vqai=hGuQ8kuc9pgc9s8qqaq=dirpe0xb9q8qiLsFr0=vr0=vr0dc8meaabaqaciaacaGaaeqabaWaaeGaeaaakeaaimaacqWFXeIraaa@3787@ ↦ Y
 MathType@MTEF@5@5@+=feaafiart1ev1aaatCvAUfKttLearuWrP9MDH5MBPbIqV92AaeXatLxBI9gBamrtHrhAL1wy0L2yHvtyaeHbnfgDOvwBHrxAJfwnaebbnrfifHhDYfgasaacH8akY=wiFfYdH8Gipec8Eeeu0xXdbba9frFj0=OqFfea0dXdd9vqai=hGuQ8kuc9pgc9s8qqaq=dirpe0xb9q8qiLsFr0=vr0=vr0dc8meaabaqaciaacaGaaeqabaWaaeGaeaaakeaaimaacqWFyeFwaaa@3851@. The goal is to find a (*θ*, *ψ*) pair that maximizes the classification accuracy.

To evaluate MR approaches, we use the well-characterized 2D HeLa set described previously [3]. The proteins in the data set were labeled using immunofluorescence, and thus, we know the ground truth, that is, which protein was labeled in each cell and subsequently imaged. This is useful for algorithm development as we can test the accuracy of classification schemes.

The challenge in this data set is that images from the same class may look different while those from different classes may look very similar (see Figure 2 in [13]). Based on the above discussion, we would like to extract discriminative features within space-frequency localized subspaces. These are obtained by MR decomposition; that is, instead of adding MR features as in [15], we compute features in the MR-decomposed subspaces. Thus, our system is a generic system with an MR decomposition block in front (see Figure 2), followed by feature computation and classification in each of the subspaces. These are then combined through a weighting process. The hypothesis we test here is that adaptive classification in MR subspaces will improve the classification accuracy.

### Base system (nMR)

We denote as *no MR *(*nMR*) the system consisting of the feature computation and the classifier blocks (see inside the dashed box in Figure 2). In our previous MR work, we used a maximum likelihood classifier that assumed the data to be well-separated Gaussian distributions, an assumption we found not to fit the data well. Instead, due to their simplicity and generality, we decided to use a two-layer neural network classifier. The first layer contains a node for each of the input features, each node using the Tan-Sigmoid transfer function. The second layer contains a node for each output and uses a linear transfer function (no hidden layers are used). We then train the neural network using a one-hot design, that is, since each output from the second layer corresponds to a class, when training, each training image will have an output of 1 for the class of which it is a member and a 0 for all other classes. To maximize the use of our data, our training process of the neural network block uses five-fold cross validation.

We ran the classifier with selected combinations of the three feature types used previously [15] (we did not use wavelet and Gabor features since they are inherently MR). These are: morphological features (*M*), Haralick texture features (*T*_1_) and Zernike moment features (*Z*). The results obtained are given in the first row of Table 1. We can see that the most powerful features on their own are texture features *T*_1_, yielding 85.49% classification accuracy. Because of that, we looked into other texture feature sets, such as the second Haralick set from [16] we termed *T*_2_, which produced a slightly better result of 85.76% (second row of Table 1). By examining these feature sets more closely, we developed a novel version of Haralick texture features, termed *T*_3 _(details are given in **Methods**). With this set, the classification accuracy of the nMR system jumped to 87.46% (third row of Table 1). We also ran the experiment for all possible combinations of feature sets, as shown in the first three rows of Table 1. We found that, while the addition of other feature sets to *T*_3 _did not increase the accuracy significantly, it did increase the number of features and thus computational complexity. This "flat" trend will turn out be general, as we will show later.

**Table 1:** Classification accuracy per class. *Z*, *M *and *T *stand for Zernike, morphological and texture features.

System	*T*	Weight.	Classification accuracy [%]						
			
			*M*	*T*	*Z*	*T, M*	*M, Z*	*T, Z*	All
nMR	*T*_1_	NW	66.12	85.49	51.20	85.76	72.48	85.06	85.04
	*T*_2_	NW	66.12	85.76	51.20	86.64	72.48	85.78	86.24
	*T*_3_	NW	66.12	**87.46**	51.20	87.38	72.48	87.12	86.86
									
MRB	*T*_3_	OF	81.62	91.82	65.42	92.04	83.38	91.66	92.36
	*T*_3_	CF	81.48	**92.32**	65.84	92.62	83.58	92.34	92.54
									
MRF	*T*_3_	OF	84.92	94.72	65.82	94.64	86.80	94.74	94.52
	*T*_3_	CF	85.16	**95.26**	65.24	**95.40**	85.88	95.26	**95.38**

**Figure 3:**
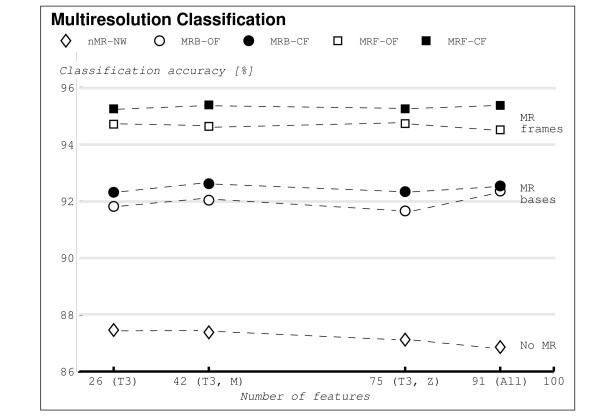
**Pictorial representation of classification accuracy results**. The diagram shows results from Table 1 for those sets involving *T*_3_, namely (*T*_3_), (*T*_3_, *M*) and (*T*_3_, *M*, *Z*). Diamond markers represent the nMR system (no MR block), circles represent the MRB system (MR bases, no redundancy) and squares represent the MRF system (MR frames, redundancy). Filled markers denote the closed-form weighting algorithm (CF), while empty ones denote the open-form weighting algorithm (OF). The following trends are noteworthy: (a) Introducing MR (both MRB and MRF) significantly outperforms nMR, thus demonstrating that classifying in MR subspaces indeed improves classification accuracy. (b) MRF outperform MRB. (b) For the two versions of the weighting algorithm, open form and closed form, the closed-form algorithm slightly outperforms the open-form one. (d) The trend in each case is almost flat across various feature set combinations, indicating that the texture set *T*_3 _alone (26 features) is sufficient for high classification accuracy.

*T*_1 _are the original Haralick texture features, *T*_2 _are modified Haralick texture features from [16] and *T*_3 _are our improved texture features. nMR denotes the base system with no MR, MRB denotes MR basis classification and MRF denotes MR frame classification. OF denotes open-form weighting algorithm while CF denotes closed-form weighting algorithm. NW denotes no weighting as there is no MR block in front. Each entry is a number denoting the classification accuracy mean over a number of trials (different orderings of the images) for a given combination of feature sets. Note that the accuracy of nMR with features *M *is the same across the rows T1, T2, T3 since texture features are not involved in the classification when morphological features alone are used (similarly with *Z*, and *M*, *Z*). A subset of these results is shown pictorially in Figure 3. These results should be compared to the best previously obtained result (on the same data set) of 91.5% [15].

### MR Bases (MRB)

We now implement our main idea of adding an MR block in front of feature computation and classification, as in Figure 2. We start with the MR decomposition being a basis expansion (details are given in **Methods**). We grow a full tree to two levels with Haar filters (see the bottom part of Figure 1). We then test the system with all feature combinations, a neural network classifier as well as two versions of the weighting algorithm (open-form and closed-form, details are given in **Methods**).

The classifier is evaluated using nested cross validations (five-fold cross validation in the neural networks block and ten-fold during the weighting process). One problem with this technique is that the initial ordering of the images determines which images are grouped together for training and testing in each fold of the cross validation. A different original ordering of the images would result in different groupings, which would be equivalent to presenting different data sets to the classifier, and would thus result in a different overall result. We solve this problem by running multiple trials, each with a random initial ordering of the images. The mean result of these trials is taken as our true classification accuracy. In our experiments, we perform ten-fold cross validation on the weight calculation.

We note the following trends: (a) For all feature combinations, MRB significantly outperforms nMR, thus demonstrating that classifying in MR subspaces indeed improves classification accuracy. (b) For the two versions of the weighting algorithm, open form and closed form, the closed-form algorithm slightly outperforms the open-form one for all feature combinations except for *M *alone (fourth and fifth rows of Table 1). In particular, for texture features *T*_3_, the accuracy rose slightly, from 91.82% to 92.32%. (c) While a slightly higher classification accuracy is obtained by using all three feature sets (92.54%) as well as both *T *and *M *(92.62%), the larger number of features and additional complexity of using *M *and *Z *features do not justify the slight improvement in accuracy (texture features *T*_3 _alone achieve 92.32%). As for nMR, this "flat" trend is good news as we can use a significantly reduced feature set and still obtain a fairly high classification accuracy.

While we were satisfied that our hypothesis seems to be true, that is, classifying in MR subspaces increases classification accuracy significantly, we decided to look more closely into how we can improve the system even more. A known issue with MRB is that they are not shift invariant (rather, they are periodically shift invariant). This is due to downsampling used and can create problems as shifted versions of data can lead to different features in MR subspaces.

Our hypothesis is that shifts in the testing set produce reduced classification accuracy. We test this hypothesis by running the algorithm with *T*_3 _features alone and with shifts of *t *= 0, 1, 2, 3 horizontally and vertically in the testing set (these shifts are chosen because we use 2 levels of the MR transform, so it is shift invariant to shifts of 2^2^*t*, but not to shifts of 2^2^*t *+ 1, 2^2^*t *+ 2, 2^2^*t *+ 3). As expected, the classification accuracy drops by 0.22%.

This experiment strongly indicates the use of MR techniques which are shift invariant (or almost shift invariant). These are called *frames *and we examine them next.

### MR Frames (MRF)

The simplest MR frame which is completely shift-invariant is called à trous [10] and is obtained by removing downsamplers (which introduce shift variance) from the scheme. This leads to redundancy but avoids the problem of shift variance. The results of the experiments with MR frames (MRF) are given in the last two rows of Table 1 (for the two versions of the weighting algorithm again).

As for MRB, the three trends are similar: (a) MRF outperforms MRB (the only set showing no improvement is *Z *alone). (b) The closed-form algorithm outperforms the open-form one whenever *T*_3 _set is involved. (c) Again, the "flat" trend continues: the difference between using *T*_3 _only as opposed to *T*_3_, *M *or all feature sets is so minor that the added number of features is not worth the complexity. We highlight these trends pictorially in Figure 3.

### Discussion and future work

Classification of protein subcellular images was indeed significantly improved by classifying in MR subspaces. One reason for this improved performance over the system using the inherently MR features is that those features are simply energies in the subbands, while here, the features can be any suitable set, leading to a more general space of solutions. A reason for the improved performance of the MR systems over the nMR one could be intuitively understood if we assumed that this data set is highly "texture"-like. For example, it is possible for two different textures to have the same set of Haralick texture features (they have the same co-occurrence matrices), while when decomposed, even at the first level, their co-occurrence matrices would be different, leading to different Haralick texture features, and thus discriminative power. An example of this is given in the compendium to the paper (see Additional file 1 and [17]).

We plan on exploring a number of issues in our future work. (a) For example, our system effectively builds an adapted MR decomposition (via subband weights) for the whole data set; we want to adapt that decomposition to each class, arguing that a different MR decomposition for a different class would be a discriminative feature in itself. We are currently working on this by adapting the closed-form algorithm. (b) We would also like to explore whether improved performance can be obtained by incorporating feature selection methods during classifier training for each subband, as was done in the original work in [15]. (c) It will also be of interest to explore how and whether to include information from parallel DNA images, since this information improved nMR-based classification accuracy in [15] from 91.5% to 92.0%. This improvement is because the parallel DNA image provides a frame of reference for distinguishing proteins that are inside or near the nucleus from those with similar patterns that are not. (d) Lastly, we would like to find a cost function that would allow us to explicitly build wavelet packets. While we implicitly do this now using weights, it would lead to improved computational efficiency if we had a method for building a subtree as opposed to using all the subbands.

## Conclusion

This paper addresses automated and robust classification of major protein subcellular location patterns. With the introduction of a multiresolution approach, we are able to obtain a high classification accuracy of 95.26% with only 26 texture features, proving that adaptive MR techniques improve the classification of the 2D HeLa data set.

## Methods

### Data set

We used the collection of 2D images of HeLa cells described previously [3] and publicly available [18]. It contains approximately 90 single-cell images of size 512 × 512, in each of *C *= 10 classes. The 10 classes of subcellular location patterns were obtained by labeling an endoplasmic reticulum protein, two Golgi proteins (giantin and gpp130), a lysosomal protein, a mitochondrial protein, a nucleolar protein, two cytoskeletal proteins (actin and tubulin), an endosomal protein, and DNA. The best previously described overall classification accuracy on this data set, without the use of the parallel DNA channel, is 91.5% [15].

### Base system (nMR)

#### Feature Sets

As in [15], we start with Haralick texture features (set *T*_1_, 13 features), morphological (set *M*, 16 features) and Zernike moments (set *Z*, 49 features). Unlike in [15], we do not use wavelet/Gabor features because the MR advantage given by these will be achieved by our MR decomposition. Therefore, our total number of features is 78, as opposed to 174 in [15].

### MR classification

We argued at the beginning of the paper that the nature of our data sets requires tools which offer localization in space and frequency as well as adaptivity, and we further argued that those tools are MR in nature. Thus, the novelty here is classifying in MR subspaces as opposed on the original image itself. The idea is that certain features will react well at a certain scale but not at another. Thus, we add an *MR *block in front of the standard feature extraction and classification blocks, as in Figure 2.

#### MR block

The basic MR block is the so-called *two-channel filter bank *(see top part of Figure 1). It, and its extensions, can be used to build decompositions custom-tailored to the image at hand. This is done by using this filter bank in a tree, iterating on any of the two-channels and its children. Moreover, the filter bank can have more than two channels, and can have more channels than the sampling factor (leading to redundant representations), etc.

Amongst the possible trees that one can use to analyze an image, the wavelet packets mentioned previously [19] adapt themselves to the signal at hand. However, this is possible only if a suitable *cost function *is available. That is, in order to adaptively build the tree, we need to find a suitable "measure" that will indicate whether a subband (a node in the tree) contains useful information or not. If it does, then we keep the node, otherwise, we prune it.

Adaptive flavors of MR have been explored for their utility in classification in various domains [20]. These studies have used the transform domain coefficients themselves as features and so had a natural cost function in selecting the tree most adapted to the signal. In [21], we used wavelet packets for fingerprint identification and verification with remarkable results.

To get a fairly general set of possibilities MR toolbox offers, we define the following matrix

*MR*_*l*,*b*,*m*,*n *_= [*ψ*_*l*,*b*,*o*,*p*_],

whose elements *ψ*_*l*,*b*,*o*,*p *_go from *o *= 0, ..., *m *- 1 and *p *= 0, ..., *n *- 1. In the above, *l *denotes the level at which the block is applied, *b *denotes the particular child branch to which it is applied, *m *denotes the number of channels and *n *the sampling factor.

If *m *= *n *for every block, the above transforms would implement a basis (nonredundant) expansion. If at least for one block *m *> *n*, the resulting decomposition is a frame and is redundant. The standard discrete dyadic wavelet transform is obtained with *m *= *n *= 2 and the MR block being applied only to the first branch of every preceding block.

As an example, we will show how our MR basis with Haar filters is represented. We have *m *= *n *= 2, and assume that we have a 1D system (a 2D system is obtained by applying the 1D one on rows and then on columns) with 1 level. Thus, *l *= 1 and the block is applied to the input branch only (we number that branch 0). The corresponding matrix is:

MR1=MR1,0,2,2=12(111−1),
 MathType@MTEF@5@5@+=feaafiart1ev1aaatCvAUfKttLearuWrP9MDH5MBPbIqV92AaeXatLxBI9gBaebbnrfifHhDYfgasaacH8akY=wiFfYdH8Gipec8Eeeu0xXdbba9frFj0=OqFfea0dXdd9vqai=hGuQ8kuc9pgc9s8qqaq=dirpe0xb9q8qiLsFr0=vr0=vr0dc8meaabaqaciaacaGaaeqabaqabeGadaaakeaacqWGnbqtcqqGGaaicqWGsbGudaWgaaWcbaGaeGymaedabeaakiabg2da9iabd2eanjabbccaGiabdkfasnaaBaaaleaacqaIXaqmcqGGSaalcqaIWaamcqGGSaalcqaIYaGmcqGGSaalcqaIYaGmaeqaaOGaeyypa0ZaaSaaaeaacqaIXaqmaeaadaGcaaqaaiabikdaYaWcbeaaaaGcdaqadaqaauaabeqaciaaaeaacqaIXaqmaeaacqaIXaqmaeaacqaIXaqmaeaacqGHsislcqaIXaqmaaaacaGLOaGaayzkaaGaeiilaWcaaa@45E1@

where the lowpass filter is given in the first column (roughly averaging) and the highpass is given in the second column (roughly differencing). This matrix describes the operation of the system on the input of length 2. Since the input sequence is in general infinite, the matrix describing the operation of the system on such a sequence is given by

MR=(⋱⋮⋮⋱⋯MR10⋯⋯0MR1⋯⋱⋮⋮⋱).
 MathType@MTEF@5@5@+=feaafiart1ev1aaatCvAUfKttLearuWrP9MDH5MBPbIqV92AaeXatLxBI9gBaebbnrfifHhDYfgasaacH8akY=wiFfYdH8Gipec8Eeeu0xXdbba9frFj0=OqFfea0dXdd9vqai=hGuQ8kuc9pgc9s8qqaq=dirpe0xb9q8qiLsFr0=vr0=vr0dc8meaabaqaciaacaGaaeqabaqabeGadaaakeaacqWGnbqtcqqGGaaicqWGsbGucqGH9aqpdaqadaqaauaabeqaeqaaaaaabaGaeSy8I8eabaGaeSO7I0eabaGaeSO7I0eabaGaeSy8I8eabaGaeS47IWeabaGaemyta0KaeeiiaaIaemOuai1aaSbaaSqaaiabigdaXaqabaaakeaacqaIWaamaeaacqWIVlctaeaacqWIVlctaeaacqaIWaamaeaacqWGnbqtcqqGGaaicqWGsbGudaWgaaWcbaGaeGymaedabeaaaOqaaiabl+UimbqaaiablgVipbqaaiabl6Uinbqaaiabl6UinbqaaiablgVipbaaaiaawIcacaGLPaaacqGGUaGlaaa@54E8@

The effect of downsampling is seen in the movement of the block *MR*_1 _by 2 each time (if the downsampling were not present, the blocks would be moving by 1).

For *l *= 2 levels, and using the same transform *MR*_1 _at each branch and at each level, we obtain the matrix *MR*_2_:

MR2=MR1⊗MR2=12(11111−11−111−1−11−1−11),
 MathType@MTEF@5@5@+=feaafiart1ev1aaatCvAUfKttLearuWrP9MDH5MBPbIqV92AaeXatLxBI9gBaebbnrfifHhDYfgasaacH8akY=wiFfYdH8Gipec8Eeeu0xXdbba9frFj0=OqFfea0dXdd9vqai=hGuQ8kuc9pgc9s8qqaq=dirpe0xb9q8qiLsFr0=vr0=vr0dc8meaabaqaciaacaGaaeqabaqabeGadaaakeaacqWGnbqtcqqGGaaicqWGsbGudaWgaaWcbaGaeGOmaidabeaakiabg2da9iabd2eanjabbccaGiabdkfasnaaBaaaleaacqaIXaqmaeqaaOGaey4LIqSaemyta0KaeeiiaaIaemOuai1aaSbaaSqaaiabikdaYaqabaGccqGH9aqpdaWcaaqaaiabigdaXaqaaiabikdaYaaadaqadaqaauaabeqaeqaaaaaabaGaeGymaedabaGaeGymaedabaGaeGymaedabaGaeGymaedabaGaeGymaedabaGaeyOeI0IaeGymaedabaGaeGymaedabaGaeyOeI0IaeGymaedabaGaeGymaedabaGaeGymaedabaGaeyOeI0IaeGymaedabaGaeyOeI0IaeGymaedabaGaeGymaedabaGaeyOeI0IaeGymaedabaGaeyOeI0IaeGymaedabaGaeGymaedaaaGaayjkaiaawMcaaiabcYcaSaaa@5685@

where ⊗ denotes the Kronecker product. The matrix describing the operation of the whole system is now an infinite block-diagonal matrix with *MR*_2 _on the diagonal (similarly to (3)).

#### Feature extraction block

Instead of combining all features into a single probability vector, we allow each feature set its own probability vector per subband. For example, for 2 levels as in (4), we have a total of 21 subbands (original image + 4 subbands at the first level + 16 subbands at the second level), effectively bringing the number of subbands to 3·*S *= 3·21 = 63 if all three feature sets are used, where *S *is the number of subbands per level. Note that although we have decreased the number of features significantly, we have also increased the number of classifiers, because we now have one classifier per subband. Evaluating this computational trade-off is a task for future work.

#### New texture feature set *T*_3_

As the Haralick texture features seem to possess the most discriminative power, we looked more closely into these. Haralick texture features are calculated using four co-occurrence matrices [16]: 1) *P*_*H *_(horizontal nearest neighbors), 2) *P*_*V *_(vertical nearest neighbors), 3) *P*_*LD *_(left diagonal nearest neighbors), and 4) *P*_*RD *_(right diagonal nearest neighbors). We now calculate 13 measures on each of these four matrices, as defined by Haralick. For example, the first two features on *P*_*H *_are:

fH,1=∑i=1Ng∑j=1Ng(PH(i,j)RH)2,
 MathType@MTEF@5@5@+=feaafiart1ev1aaatCvAUfKttLearuWrP9MDH5MBPbIqV92AaeXatLxBI9gBaebbnrfifHhDYfgasaacH8akY=wiFfYdH8Gipec8Eeeu0xXdbba9frFj0=OqFfea0dXdd9vqai=hGuQ8kuc9pgc9s8qqaq=dirpe0xb9q8qiLsFr0=vr0=vr0dc8meaabaqaciaacaGaaeqabaqabeGadaaakeaacqWGMbGzdaWgaaWcbaGaemisaGKaeiilaWIaeGymaedabeaakiabg2da9maaqahabaWaaabCaeaadaqadaqaamaalaaabaGaemiuaa1aaSbaaSqaaiabdIeaibqabaGccqGGOaakcqWGPbqAcqGGSaalcqWGQbGAcqGGPaqkaeaacqWGsbGudaWgaaWcbaGaemisaGeabeaaaaaakiaawIcacaGLPaaaaSqaaiabdQgaQjabg2da9iabigdaXaqaaiabd6eaonaaBaaameaacqWGNbWzaeqaaaqdcqGHris5aaWcbaGaemyAaKMaeyypa0JaeGymaedabaGaemOta40aaSbaaWqaaiabdEgaNbqabaaaniabggHiLdGcdaahaaWcbeqaaiabikdaYaaakiabcYcaSaaa@508A@

fH,2=∑k=0Ng−1k2∑|i−j|=kPH(i,j)RH,
 MathType@MTEF@5@5@+=feaafiart1ev1aaatCvAUfKttLearuWrP9MDH5MBPbIqV92AaeXatLxBI9gBaebbnrfifHhDYfgasaacH8akY=wiFfYdH8Gipec8Eeeu0xXdbba9frFj0=OqFfea0dXdd9vqai=hGuQ8kuc9pgc9s8qqaq=dirpe0xb9q8qiLsFr0=vr0=vr0dc8meaabaqaciaacaGaaeqabaqabeGadaaakeaacqWGMbGzdaWgaaWcbaGaemisaGKaeiilaWIaeGOmaidabeaakiabg2da9maaqahabaGaem4AaS2aaWbaaSqabeaacqaIYaGmaaaabaGaem4AaSMaeyypa0JaeGimaadabaGaemOta40aaSbaaWqaaiabdEgaNbqabaWccqGHsislcqaIXaqma0GaeyyeIuoakmaaqafabaWaaSaaaeaacqWGqbaudaWgaaWcbaGaemisaGeabeaakiabcIcaOiabdMgaPjabcYcaSiabdQgaQjabcMcaPaqaaiabdkfasnaaBaaaleaacqWGibasaeqaaaaaaeaacqGG8baFcqWGPbqAcqGHsislcqWGQbGAcqGG8baFcqGH9aqpcqWGRbWAaeqaniabggHiLdGccqGGSaalaaa@551B@

where *N*_*g *_is the number of gray levels in the image and *R*_*H *_is a normalizing constant equal to the sum of all the elements in *P*_*H*_.

The other measures follow in similar fashion, giving us four sets of 13 measures: *f*_(*H*, 1–13), _*f*_(*V*, 1–13), _*f*_(*LD*, 1–13) _and *f*_(*RD*, 1–13)_. Haralick's original method reduces these to a single set of 13 by calculating the mean of each measure across the four sets (feature set *T*_1_):

fi(T1)=fH,i+fV,i+fLD,i+fRD,i4,
 MathType@MTEF@5@5@+=feaafiart1ev1aaatCvAUfKttLearuWrP9MDH5MBPbIqV92AaeXatLxBI9gBaebbnrfifHhDYfgasaacH8akY=wiFfYdH8Gipec8Eeeu0xXdbba9frFj0=OqFfea0dXdd9vqai=hGuQ8kuc9pgc9s8qqaq=dirpe0xb9q8qiLsFr0=vr0=vr0dc8meaabaqaciaacaGaaeqabaqabeGadaaakeaacqWGMbGzdaqhaaWcbaGaemyAaKgabaGaeiikaGIaemivaq1aaSbaaWqaaiabigdaXaqabaWccqGGPaqkaaGccqGH9aqpdaWcaaqaaiabdAgaMnaaBaaaleaacqWGibascqGGSaalcqWGPbqAaeqaaOGaey4kaSIaemOzay2aaSbaaSqaaiabdAfawjabcYcaSiabdMgaPbqabaGccqGHRaWkcqWGgbGrdaWgaaWcbaGaemitaWKaemiraqKaeiilaWIaemyAaKgabeaakiabgUcaRiabdAgaMnaaBaaaleaacqWGsbGucqWGebarcqGGSaalcqWGPbqAaeqaaaGcbaGaeGinaqdaaiabcYcaSaaa@4EC6@

for *i *= 1, . . ., 13. An alternative method [16] that we have used previously [22], is to use both the mean and the range of the 13 measures, thus resulting in two sets of 13 features (26 features overall, feature set *T*_2_). 

We can significantly improve upon these results by making a change in the way that we combine our initial four sets of features. We note that *P*_*H *_and *P*_*V *_are fundamentally different from *P*_*LD *_and *P*_*HD *_because adjacent neighboring pixels are spatially closer than diagonal neighboring pixels. Therefore, instead of averaging the features from all four sets, we create our first set of 13 features by averaging *f*_(*H*,*i*) _and *f*_(*V*,*i*)_, and a second set of 13 features by averaging *f*_(*LD*,*i*) _and *f*_(*RD*,*i*)_. Thus, we end up with two sets of 13 features, which are concatenated into one feature set (*T*_3_) of 26 features:

fi(T3)=fH,i+fV,i2,fi+13(T3)=fLD,i+fRD,i2,
 MathType@MTEF@5@5@+=feaafiart1ev1aaatCvAUfKttLearuWrP9MDH5MBPbIqV92AaeXatLxBI9gBaebbnrfifHhDYfgasaacH8akY=wiFfYdH8Gipec8Eeeu0xXdbba9frFj0=OqFfea0dXdd9vqai=hGuQ8kuc9pgc9s8qqaq=dirpe0xb9q8qiLsFr0=vr0=vr0dc8meaabaqaciaacaGaaeqabaqabeGadaaakeaafaqabeqacaaabaGaemOzay2aa0baaSqaaiabdMgaPbqaaiabcIcaOiabdsfaunaaBaaameaacqaIZaWmaeqaaSGaeiykaKcaaOGaeyypa0ZaaSaaaeaacqWGMbGzdaWgaaWcbaGaemisaGKaeiilaWIaemyAaKgabeaakiabgUcaRiabdAgaMnaaBaaaleaacqWGwbGvcqGGSaalcqWGPbqAaeqaaaGcbaGaeGOmaidaaiabcYcaSaqaaiabdAgaMnaaDaaaleaacqWGPbqAcqGHRaWkcqaIXaqmcqaIZaWmaeaacqGGOaakcqWGubavdaWgaaadbaGaeG4mamdabeaaliabcMcaPaaakiabg2da9maalaaabaGaemOzay2aaSbaaSqaaiabdYeamjabdseaejabcYcaSiabdMgaPbqabaGccqGHRaWkcqWGMbGzdaWgaaWcbaGaemOuaiLaemiraqKaeiilaWIaemyAaKgabeaaaOqaaiabikdaYaaacqGGSaalaaaaaa@5AD5@

for *i *= 1, ..., 13.

#### Weighting algorithms

Figure 2 shows a graphical representation of a generic MR classification system, including the process of combining all of the subband decisions into one. We use weights for each subband to adjust the importance that a particular subband has on the overall decision made by the classification system. If the weights are chosen such that the no decomposition weight is equal to 1, and all other weights are 0, we will achieve the same output vector as we would have without using the adaptive MR system. Therefore, we know that there exists a weight combination that will do at least as well as the generic classifier (when no MR is involved) in the training phase. Our goal is to decide how to find the weight vector that achieves the highest overall classification accuracy on a given data set. We developed two versions of the weighting algorithm: open-form and closed-form.

The difference between the open- and closed-form algorithms is that in the open-form version we optimize classification accuracy on the training set as opposed to the closed-form where we look for the least-squares solution. The open-form algorithm for the training and the testing phases are given in [13] under Algorithms 1 and 2, respectively.

The neural network block outputs a series of decision vectors for each subband of each training image. Each decision vector ds(r)
 MathType@MTEF@5@5@+=feaafiart1ev1aaatCvAUfKttLearuWrP9MDH5MBPbIqV92AaeXatLxBI9gBaebbnrfifHhDYfgasaacH8akY=wiFfYdH8Gipec8Eeeu0xXdbba9frFj0=OqFfea0dXdd9vqai=hGuQ8kuc9pgc9s8qqaq=dirpe0xb9q8qiLsFr0=vr0=vr0dc8meaabaqaciaacaGaaeqabaqabeGadaaakeaacqWGKbazdaqhaaWcbaGaem4CamhabaGaeiikaGIaemOCaiNaeiykaKcaaaaa@32B8@ contains *C *= 10 numbers (because we have 10 classes) that correspond to the "local" decisions made by the subband *s *for a specific image *r*.

#### Open-form algorithm

If using the open-form algorithm, we initialize all the weights (see Algorithm 1 in [13] for details), and a global decision vector is computed using a weighted sum of the local decisions. An initial class label will be given to an image using this global decision vector. If that class label is correct, we go to the next image. If it is incorrect, we look at the local decisions of each subband and adjust the weights of each subband *s *as follows:

wsiter={wsiter−1⋅(1+ε)if subband s is correct,wsiter−1⋅(1−ε)otherwise.
 MathType@MTEF@5@5@+=feaafiart1ev1aaatCvAUfKttLearuWrP9MDH5MBPbIqV92AaeXatLxBI9gBaebbnrfifHhDYfgasaacH8akY=wiFfYdH8Gipec8Eeeu0xXdbba9frFj0=OqFfea0dXdd9vqai=hGuQ8kuc9pgc9s8qqaq=dirpe0xb9q8qiLsFr0=vr0=vr0dc8meaabaqaciaacaGaaeqabaqabeGadaaakeaacqWG3bWDdaqhaaWcbaGaem4CamhabaGaemyAaKMaemiDaqNaemyzauMaemOCaihaaOGaeyypa0ZaaiqabeaafaqaaeGacaaabaGaem4DaC3aa0baaSqaaiabdohaZbqaaiabdMgaPjabdsha0jabdwgaLjabdkhaYjabgkHiTiabigdaXaaakiabgwSixlabcIcaOiabigdaXiabgUcaRGGaciab=v7aLjabcMcaPaqaaiabbMgaPjabbAgaMjabbccaGiabbohaZjabbwha1jabbkgaIjabbkgaIjabbggaHjabb6gaUjabbsgaKjabbccaGiabdohaZjabbccaGiabbMgaPjabbohaZjabbccaGiabbogaJjabb+gaVjabbkhaYjabbkhaYjabbwgaLjabbogaJjabbsha0jabcYcaSaqaaiabdEha3naaDaaaleaacqWGZbWCaeaacqWGPbqAcqWG0baDcqWGLbqzcqWGYbGCcqGHsislcqaIXaqmaaGccqGHflY1cqGGOaakcqaIXaqmcqGHsislcqWF1oqzcqGGPaqkaeaacqqGVbWBcqqG0baDcqqGObaAcqqGLbqzcqqGYbGCcqqG3bWDcqqGPbqAcqqGZbWCcqqGLbqzcqGGUaGlaaaacaGL7baaaaa@86C0@

where *iter *is the iteration number and *ε *is a small positive constant. This can be viewed as a reward/punishment method where the subbands taking the correct decisions will have their weights increased, and those taking wrong decisions will have their weights decreased. We continue cycling through the images until there is no increase in classification accuracy on the training set for a given number of iterations.

#### Closed-form algorithm

The closed-form solution does not use an iterative algorithm. Instead, it finds the weight vector by solving a minimization problem in the least-square sense. We now explain how this is accomplished.

Assume that we have *R *training images. For each training image *r *= 1, . . ., *R*, the vector ds(r)=(ds,1(r),ds,2(r),...,ds,C(r))T
 MathType@MTEF@5@5@+=feaafiart1ev1aaatCvAUfKttLearuWrP9MDH5MBPbIqV92AaeXatLxBI9gBaebbnrfifHhDYfgasaacH8akY=wiFfYdH8Gipec8Eeeu0xXdbba9frFj0=OqFfea0dXdd9vqai=hGuQ8kuc9pgc9s8qqaq=dirpe0xb9q8qiLsFr0=vr0=vr0dc8meaabaqaciaacaGaaeqabaqabeGadaaakeaacqWGKbazdaqhaaWcbaGaem4CamhabaGaeiikaGIaemOCaiNaeiykaKcaaOGaeyypa0JaeiikaGIaemizaq2aa0baaSqaaiabdohaZjabcYcaSiabigdaXaqaaiabcIcaOiabdkhaYjabcMcaPaaakiabcYcaSiabdsgaKnaaDaaaleaacqWGZbWCcqGGSaalcqaIYaGmaeaacqGGOaakcqWGYbGCcqGGPaqkaaGccqGGSaalcqGGUaGlcqGGUaGlcqGGUaGlcqGGSaalcqWGKbazdaqhaaWcbaGaem4CamNaeiilaWIaem4qameabaGaeiikaGIaemOCaiNaeiykaKcaaOGaeiykaKYaaWbaaSqabeaacqWGubavaaaaaa@53F7@, is the *C *× 1 decision vector at the output of each subband classifier *s*, where ds,c(r)
 MathType@MTEF@5@5@+=feaafiart1ev1aaatCvAUfKttLearuWrP9MDH5MBPbIqV92AaeXatLxBI9gBaebbnrfifHhDYfgasaacH8akY=wiFfYdH8Gipec8Eeeu0xXdbba9frFj0=OqFfea0dXdd9vqai=hGuQ8kuc9pgc9s8qqaq=dirpe0xb9q8qiLsFr0=vr0=vr0dc8meaabaqaciaacaGaaeqabaqabeGadaaakeaacqWGKbazdaqhaaWcbaGaem4CamNaeiilaWIaem4yamgabaGaeiikaGIaemOCaiNaeiykaKcaaaaa@34E7@ indicates the confidence of subband *s *that the training image *r *belongs to class *c*. For each training image *r*, the weighting block takes as input the subband (local) decision vectors ds(r)
 MathType@MTEF@5@5@+=feaafiart1ev1aaatCvAUfKttLearuWrP9MDH5MBPbIqV92AaeXatLxBI9gBaebbnrfifHhDYfgasaacH8akY=wiFfYdH8Gipec8Eeeu0xXdbba9frFj0=OqFfea0dXdd9vqai=hGuQ8kuc9pgc9s8qqaq=dirpe0xb9q8qiLsFr0=vr0=vr0dc8meaabaqaciaacaGaaeqabaqabeGadaaakeaacqWGKbazdaqhaaWcbaGaem4CamhabaGaeiikaGIaemOCaiNaeiykaKcaaaaa@32B8@ and combines them into a single output decision vector as follows:

∑s=1Swsds(r).
 MathType@MTEF@5@5@+=feaafiart1ev1aaatCvAUfKttLearuWrP9MDH5MBPbIqV92AaeXatLxBI9gBaebbnrfifHhDYfgasaacH8akY=wiFfYdH8Gipec8Eeeu0xXdbba9frFj0=OqFfea0dXdd9vqai=hGuQ8kuc9pgc9s8qqaq=dirpe0xb9q8qiLsFr0=vr0=vr0dc8meaabaqaciaacaGaaeqabaqabeGadaaakeaadaaeWbqaaiabdEha3naaBaaaleaacqWGZbWCaeqaaOGaemizaq2aa0baaSqaaiabdohaZbqaaiabcIcaOiabdkhaYjabcMcaPaaaaeaacqWGZbWCcqGH9aqpcqaIXaqmaeaacqWGtbWua0GaeyyeIuoakiabc6caUaaa@3D8D@

We can rewrite the above by, for each training image *r*, forming a matrix *D*^(*r*) ^of size *C *× *S*, where each element Dc,s(r)
 MathType@MTEF@5@5@+=feaafiart1ev1aaatCvAUfKttLearuWrP9MDH5MBPbIqV92AaeXatLxBI9gBaebbnrfifHhDYfgasaacH8akY=wiFfYdH8Gipec8Eeeu0xXdbba9frFj0=OqFfea0dXdd9vqai=hGuQ8kuc9pgc9s8qqaq=dirpe0xb9q8qiLsFr0=vr0=vr0dc8meaabaqaciaacaGaaeqabaqabeGadaaakeaacqWGebardaqhaaWcbaGaem4yamMaeiilaWIaem4CamhabaGaeiikaGIaemOCaiNaeiykaKcaaaaa@34A7@ is the value at position *c *of the decision vector ds(r)
 MathType@MTEF@5@5@+=feaafiart1ev1aaatCvAUfKttLearuWrP9MDH5MBPbIqV92AaeXatLxBI9gBaebbnrfifHhDYfgasaacH8akY=wiFfYdH8Gipec8Eeeu0xXdbba9frFj0=OqFfea0dXdd9vqai=hGuQ8kuc9pgc9s8qqaq=dirpe0xb9q8qiLsFr0=vr0=vr0dc8meaabaqaciaacaGaaeqabaqabeGadaaakeaacqWGKbazdaqhaaWcbaGaem4CamhabaGaeiikaGIaemOCaiNaeiykaKcaaaaa@32B8@ of subband classifier *s*. We can now compute:

*D*^(*r*)^*w*,

where *w *= (*w*_1_, . . ., *w*_*S*_)^*T *^is of size *S *× 1. Thus, we want to find a weight vector *w *common to all training images *r *= 1, . . ., *R*. A possible solution for *w *is the one that minimizes the error in the least-square sense:

wwin=arg⁡min⁡w∑r=1R||d(r)−D(r)w||2,
 MathType@MTEF@5@5@+=feaafiart1ev1aaatCvAUfKttLearuWrP9MDH5MBPbIqV92AaeXatLxBI9gBaebbnrfifHhDYfgasaacH8akY=wiFfYdH8Gipec8Eeeu0xXdbba9frFj0=OqFfea0dXdd9vqai=hGuQ8kuc9pgc9s8qqaq=dirpe0xb9q8qiLsFr0=vr0=vr0dc8meaabaqaciaacaGaaeqabaqabeGadaaakeaacqWG3bWDdaWgaaWcbaGaem4DaCNaemyAaKMaemOBa4gabeaakiabg2da9iGbcggaHjabckhaYjabcEgaNnaaxababaGagiyBa0MaeiyAaKMaeiOBa4galeaacqWG3bWDaeqaaOWaaabCaeaacqGG8baFcqGG8baFcqWGKbazdaahaaWcbeqaaiabcIcaOiabdkhaYjabcMcaPaaakiabgkHiTiabdseaenaaCaaaleqabaGaeiikaGIaemOCaiNaeiykaKcaaOGaem4DaCNaeiiFaWNaeiiFaW3aaWbaaSqabeaacqaIYaGmaaaabaGaemOCaiNaeyypa0JaeGymaedabaGaemOuaifaniabggHiLdGccqGGSaalaaa@57C2@

where *d*^(*r*) ^is the desired target decision vector of size *C *× 1. It has a 1 in the position of the true class, and 0 elsewhere.

We need to rewrite the above in a direct error-minimization form. We thus define a target output vector *d *of size *CR *× 1, as a vector which concatenates all the target decision vectors *d*^(*r*) ^as follows:

d=(d1(1), d2(1), ..., dC(1)︸training image 1, ..., d1(R),..., dC(R)︸training image R)T,
 MathType@MTEF@5@5@+=feaafiart1ev1aaatCvAUfKttLearuWrP9MDH5MBPbIqV92AaeXatLxBI9gBaebbnrfifHhDYfgasaacH8akY=wiFfYdH8Gipec8Eeeu0xXdbba9frFj0=OqFfea0dXdd9vqai=hGuQ8kuc9pgc9s8qqaq=dirpe0xb9q8qiLsFr0=vr0=vr0dc8meaabaqaciaacaGaaeqabaqabeGadaaakeaacqWGKbazcqGH9aqpdaqadaqaamaayaaabaGaemizaq2aa0baaSqaaiabigdaXaqaaiabcIcaOiabigdaXiabcMcaPaaakiabcYcaSiabdsgaKnaaDaaaleaacqaIYaGmaeaacqGGOaakcqaIXaqmcqGGPaqkaaGccqGGSaalcqGGUaGlcqGGUaGlcqGGUaGlcqGGSaalcqWGKbazdaqhaaWcbaGaem4qameabaGaeiikaGIaeGymaeJaeiykaKcaaaqaaiabbsha0jabbkhaYjabbggaHjabbMgaPjabb6gaUjabbMgaPjabb6gaUjabbEgaNjabbccaGiabbMgaPjabb2gaTjabbggaHjabbEgaNjabbwgaLjabbccaGiabigdaXaGccaGL44pacqGGSaalcqGGUaGlcqGGUaGlcqGGUaGlcqGGSaaldaagaaqaaiabdsgaKnaaDaaaleaacqaIXaqmaeaacqGGOaakcqWGsbGucqGGPaqkaaGccqGGSaalcqGGUaGlcqGGUaGlcqGGUaGlcqGGSaalcqWGKbazdaqhaaWcbaGaem4qameabaGaeiikaGIaemOuaiLaeiykaKcaaaqaaiabbsha0jabbkhaYjabbggaHjabbMgaPjabb6gaUjabbMgaPjabb6gaUjabbEgaNjabbccaGiabbMgaPjabb2gaTjabbggaHjabbEgaNjabbwgaLjabbccaGiabdkfasbGccaGL44paaiaawIcacaGLPaaadaahaaWcbeqaaiabdsfaubaakiabcYcaSaaa@8775@

and a *CR *× *S *matrix *D *consisting of the all the decision matrices *D*^(*r*) ^of all the training data stacked on top of each other:

D=(D1,1(1)⋯D1,S(1)⋮⋱⋮DC,1(1)⋯DC,S(1)⋮⋱⋮D1,1(R)⋯D1,S(R)⋮⋱⋮DC,1(R)⋯DC,S(R)).
 MathType@MTEF@5@5@+=feaafiart1ev1aaatCvAUfKttLearuWrP9MDH5MBPbIqV92AaeXatLxBI9gBaebbnrfifHhDYfgasaacH8akY=wiFfYdH8Gipec8Eeeu0xXdbba9frFj0=OqFfea0dXdd9vqai=hGuQ8kuc9pgc9s8qqaq=dirpe0xb9q8qiLsFr0=vr0=vr0dc8meaabaqaciaacaGaaeqabaqabeGadaaakeaacqWGebarcqGH9aqpdaqadaqaauaabeqahmaaaaqaaiabdseaenaaDaaaleaacqaIXaqmcqGGSaalcqaIXaqmaeaacqGGOaakcqaIXaqmcqGGPaqkaaaakeaacqWIVlctaeaacqWGebardaqhaaWcbaGaeGymaeJaeiilaWIaem4uamfabaGaeiikaGIaeGymaeJaeiykaKcaaaGcbaGaeSO7I0eabaGaeSy8I8eabaGaeSO7I0eabaGaemiraq0aa0baaSqaaiabdoeadjabcYcaSiabigdaXaqaaiabcIcaOiabigdaXiabcMcaPaaaaOqaaiabl+UimbqaaiabdseaenaaDaaaleaacqWGdbWqcqGGSaalcqWGtbWuaeaacqGGOaakcqaIXaqmcqGGPaqkaaaakeaacqWIUlstaeaacqWIXlYtaeaacqWIUlstaeaacqWGebardaqhaaWcbaGaeGymaeJaeiilaWIaeGymaedabaGaeiikaGIaemOuaiLaeiykaKcaaaGcbaGaeS47IWeabaGaemiraq0aa0baaSqaaiabigdaXiabcYcaSiabdofatbqaaiabcIcaOiabdkfasjabcMcaPaaaaOqaaiabl6UinbqaaiablgVipbqaaiabl6UinbqaaiabdseaenaaDaaaleaacqWGdbWqcqGGSaalcqaIXaqmaeaacqGGOaakcqWGsbGucqGGPaqkaaaakeaacqWIVlctaeaacqWGebardaqhaaWcbaGaem4qamKaeiilaWIaem4uamfabaGaeiikaGIaemOuaiLaeiykaKcaaaaaaOGaayjkaiaawMcaaiabc6caUaaa@8238@

We can now rewrite (7) as:

wwin=arg⁡min⁡w‖d−Dw‖,
 MathType@MTEF@5@5@+=feaafiart1ev1aaatCvAUfKttLearuWrP9MDH5MBPbIqV92AaeXatLxBI9gBaebbnrfifHhDYfgasaacH8akY=wiFfYdH8Gipec8Eeeu0xXdbba9frFj0=OqFfea0dXdd9vqai=hGuQ8kuc9pgc9s8qqaq=dirpe0xb9q8qiLsFr0=vr0=vr0dc8meaabaqaciaacaGaaeqabaqabeGadaaakeaacqWG3bWDdaWgaaWcbaGaem4DaCNaemyAaKMaemOBa4gabeaakiabg2da9iGbcggaHjabckhaYjabcEgaNnaaxababaGagiyBa0MaeiyAaKMaeiOBa4galeaacqWG3bWDaeqaaOWaauWaaeaacqWGKbazcqGHsislcqWGebarcqWG3bWDaiaawMa7caGLkWoacqGGSaalaaa@464D@

which possesses a closed-form solution and can be computed efficiently.

Then, for a testing image *t*, we compute its decision vector *δ *= (*δ*_1_, *δ*_2_, . . ., *δ*_*C*_) as follows:

δ=∑s=1Swwin,sds(t),
 MathType@MTEF@5@5@+=feaafiart1ev1aaatCvAUfKttLearuWrP9MDH5MBPbIqV92AaeXatLxBI9gBaebbnrfifHhDYfgasaacH8akY=wiFfYdH8Gipec8Eeeu0xXdbba9frFj0=OqFfea0dXdd9vqai=hGuQ8kuc9pgc9s8qqaq=dirpe0xb9q8qiLsFr0=vr0=vr0dc8meaabaqaciaacaGaaeqabaqabeGadaaakeaaiiGacqWF0oazcqGH9aqpdaaeWbqaaiabdEha3naaBaaaleaacqWG3bWDcqWGPbqAcqWGUbGBcqGGSaalcqWGZbWCaeqaaOGaemizaq2aa0baaSqaaiabdohaZbqaaiabcIcaOiabdsha0jabcMcaPaaaaeaacqWGZbWCcqGH9aqpcqaIXaqmaeaacqWGtbWua0GaeyyeIuoakiabcYcaSaaa@4556@

where ds(t)
 MathType@MTEF@5@5@+=feaafiart1ev1aaatCvAUfKttLearuWrP9MDH5MBPbIqV92AaeXatLxBI9gBaebbnrfifHhDYfgasaacH8akY=wiFfYdH8Gipec8Eeeu0xXdbba9frFj0=OqFfea0dXdd9vqai=hGuQ8kuc9pgc9s8qqaq=dirpe0xb9q8qiLsFr0=vr0=vr0dc8meaabaqaciaacaGaaeqabaqabeGadaaakeaacqWGKbazdaqhaaWcbaGaem4CamhabaGaeiikaGIaemiDaqNaeiykaKcaaaaa@32BC@ are the local decision vectors for *t*. The classification decision is then made as

cwin=arg⁡max⁡cδc,
 MathType@MTEF@5@5@+=feaafiart1ev1aaatCvAUfKttLearuWrP9MDH5MBPbIqV92AaeXatLxBI9gBaebbnrfifHhDYfgasaacH8akY=wiFfYdH8Gipec8Eeeu0xXdbba9frFj0=OqFfea0dXdd9vqai=hGuQ8kuc9pgc9s8qqaq=dirpe0xb9q8qiLsFr0=vr0=vr0dc8meaabaqaciaacaGaaeqabaqabeGadaaakeaacqWGJbWydaWgaaWcbaGaem4DaCNaemyAaKMaemOBa4gabeaakiabg2da9iGbcggaHjabckhaYjabcEgaNnaaxababaGagiyBa0MaeiyyaeMaeiiEaGhaleaacqWGJbWyaeqaaGGacOGae8hTdq2aaSbaaSqaaiabdogaJbqabaGccqGGSaalaaa@4145@

that is, the winning class corresponds to the index of the highest coefficient in *δ*.

### MR Bases

Among all possible combinations given in (1), we now confine ourselves to those implementing bases, that is, the resulting decompositions are nonredundant. Thus, in each MR subblock, *m *= *n*.

We grow a full MR tree with 2 levels. The classification system uses all the subspaces from the root (the original image) to the leaves of the tree. Hence, the total number of subbands used is 21 (1 + 4 + 4^2^). We used the simplest, Haar filters in the decomposition, where the lowpass is given by g=12(1,1)T
 MathType@MTEF@5@5@+=feaafiart1ev1aaatCvAUfKttLearuWrP9MDH5MBPbIqV92AaeXatLxBI9gBaebbnrfifHhDYfgasaacH8akY=wiFfYdH8Gipec8Eeeu0xXdbba9frFj0=OqFfea0dXdd9vqai=hGuQ8kuc9pgc9s8qqaq=dirpe0xb9q8qiLsFr0=vr0=vr0dc8meaabaqaciaacaGaaeqabaqabeGadaaakeaacqWGNbWzcqGH9aqpdaWcaaqaaiabigdaXaqaamaakaaabaGaeGOmaidaleqaaaaakiabcIcaOiabigdaXiabcYcaSiabigdaXiabcMcaPmaaCaaaleqabaGaeSy==7gaaaaa@38BA@ whereas the highpass is h=12(1,−1)T
 MathType@MTEF@5@5@+=feaafiart1ev1aaatCvAUfKttLearuWrP9MDH5MBPbIqV92AaeXatLxBI9gBaebbnrfifHhDYfgasaacH8akY=wiFfYdH8Gipec8Eeeu0xXdbba9frFj0=OqFfea0dXdd9vqai=hGuQ8kuc9pgc9s8qqaq=dirpe0xb9q8qiLsFr0=vr0=vr0dc8meaabaqaciaacaGaaeqabaqabeGadaaakeaacqWGObaAcqGH9aqpdaWcaaqaaiabigdaXaqaamaakaaabaGaeGOmaidaleqaaaaakiabcIcaOiabigdaXiabcYcaSiabgkHiTiabigdaXiabcMcaPmaaCaaaleqabaGaeSy==7gaaaaa@39A9@. Given a 1D input sequence *x*, the MR transform we apply to each block of 4 elements (advancing each time by 4) is given by the matrix defined in (4). This is done first in the horizontal direction and then in the vertical one, producing 16 outputs (subbands). There are many other MRB blocks possible, the investigation of which is left for future work.

### MR Frames

We now lift the restriction of no redundancy and allow *m *and *n *to be different (*m *> *n*). The resulting decompositions are called frames [23].

We use again the full MR tree with 2 levels, but remove downsamplers, as in the à trous algorithm [10]. Given a 1D input sequence *x*, the MR transform we apply to each block of 4 elements is identical to the one in (4) except that it is applied to every block of 4 elements (there is no downsampling). There are many other MRF blocks possible, the investigation of which is left for future work.

## Reproducible research

All the material needed to reproduce results in this paper is available at the web site address [17] and provided in the Additional file 1 as well.

## Authors' contributions

AC coordinated the work, helped conceive the approach and helped extensively with writing the manuscript. YB was in charge of unifying the code and designing and running the experiments involving multiresolution and frames. CJ designed new texture features and helped extensively with writing the manuscript. TM conceived the enhanced multiresolution approach. GS was involved in the original multiresolution approach to classification, the discussions throughout, and helped extensively with writing the manuscript. RFM helped throughout with discussions and editing the manuscript. JK conceived the multiresolution approach and was fully involved in every step of the work, from designing the experiments through interpretation as well as drafting the manuscript. All of the authors read and approved the final manuscript.

## Supplementary Material

Additional file 1 Compendium. 07_ChebiraBJMSMK_compendium.zip. This file is a compressed archive that contains the code that generated the results in this paper, the pseudo-code for the weighting algorithms, Table 1 with detailed results and index files of the web site containing all of this material [17]. Click here for file
