# Supplementary material for: A multiresolution approach to automated classification of protein subcellular location images
Source: BMC Bioinformatics. 2007 Jun 19;8:210. doi: 10.1186/1471-2105-8-210 (PMC1933440; doi:10.1186/1471-2105-8-210)
Supplement: Additional file 1 — Compendium. 07_ChebiraBJMSMK_compendium.zip. This file is a compressed archive that contains the code that generated the results in this paper, the pseudo-code for the weighting algorithms, Table 1 with detailed results and index files of the web site containing all of this material [17]. [file 1471-2105-8-210-S1.zip › 07_ChebiraMSBJK_code/lib/m2html/templates/blue/mdir.tpl]

Index for Directory {MDIR}


|  |  |
| --- | --- |
| Master index | Index for {MDIR} |

# Index for {MDIR}

## Matlab files in this directory:

|  |  |
| --- | --- |
| {NAME} | {H1LINE} |

## Other Matlab-specific files in this directory:

- {OTHERFILE}


## Subsequent directories:

- {SUBDIRECTORY}


## Dependency Graph

- View the Graph.


## TODO List

- View the TODO list.


---

Generated on {DATE} by **m2html** © 2003
