# Supplementary material for: A multiresolution approach to automated classification of protein subcellular location images
Source: BMC Bioinformatics. 2007 Jun 19;8:210. doi: 10.1186/1471-2105-8-210 (PMC1933440; doi:10.1186/1471-2105-8-210)
Supplement: Additional file 1 — Compendium. 07_ChebiraBJMSMK_compendium.zip. This file is a compressed archive that contains the code that generated the results in this paper, the pseudo-code for the weighting algorithms, Table 1 with detailed results and index files of the web site containing all of this material [17]. [file 1471-2105-8-210-S1.zip › 07_ChebiraMSBJK_code/lib/m2html/templates/blue/todo.tpl]

To Do List for {MDIR}


|  |  |
| --- | --- |
| Master index | Index for {MDIR} |

# To Do List for {MDIR}

## {MFILE}:

- line {NBLINE}:  {COMMENT}


---

Generated on {DATE} by **m2html** © 2003
