# Supplementary material for: A multiresolution approach to automated classification of protein subcellular location images
Source: BMC Bioinformatics. 2007 Jun 19;8:210. doi: 10.1186/1471-2105-8-210 (PMC1933440; doi:10.1186/1471-2105-8-210)
Supplement: Additional file 1 — Compendium. 07_ChebiraBJMSMK_compendium.zip. This file is a compressed archive that contains the code that generated the results in this paper, the pseudo-code for the weighting algorithms, Table 1 with detailed results and index files of the web site containing all of this material [17]. [file 1471-2105-8-210-S1.zip › index.html]

Chebira et al., A multiresolution approach to automated classificaiton
of protein subcellular location images


|  |  |  |
| --- | --- | --- |
| **<** | bimagicLab | **>** |
|  | **b****io****imag****e** **i****nformati****c****s lab** |  |

 

---

Jelena Kovačević
| bimagicLab

---

|  |
| --- |
| **Compendium**  ---  Paper information and status  A. Chebira, Y. Barbotin, C. Jackson, T.E. Merryman, G. Srinivasa, R. F. Murphy and J. Kovačević, “A multiresolution approach to automated classification of protein subcellular location images”, BMC Bioinformatics, 2007.  Abstract  Background: The problem of automated interpretation of fluorescence microscope images depicting subcellular protein locations is at the forefront of the current trend in biology towards understanding the role and function of all proteins. Over the past ten years, the feasibility of using machine learning methods to recognize all major subcellular location patterns has been convincingly demonstrated, using diverse feature sets and combinations of classifiers.  On a well-studied data set of 2D HeLa single-cell images, the best performance to date, 91.5%, was obtained upon the addition of a simple set of multiresolution features.     Results: We report here a novel approach for the classification of subcellular location patterns by classifying in multiresolution subspaces. Our system is able to work with any feature set and any classifier. It consists of multiresolution (MR) decomposition, followed by feature computation and classification in each MR subspace, yielding local decisions that are then combined into a global decision.  With 26 texture features alone and a neural network classifier, we obtained an increase in accuracy on the 2D HeLa data set to 95.3%.     Conclusions: We demonstrate that the space-frequency localized information in the multiresolution subspaces adds significantly to the discriminative power of the system. Moreover, we show that a vastly reduced set of features is sufficient, consisting of our novel modified Haralick texture features. Our proposed system is general, allowing for any combinations of sets of features and any combination of classifiers.  Data  2D and 3D HeLa data sets available from MurphyLab.  Code  Readme file as well as the code to generate all the figures and tables in the paper.  [07\_ChebiraMSBJK\_code directory]  Pseudo-code  Pseudo code for the algorithms in the paper.  [pdf]  Proofs  NA  Other material  Table 1 with variances included.  [pdf]  List of tested configurations  Matlab 7.0.1 on Linux (Rocks)  For more information or to report bugs  achebira@andrew.cmu.edu |
|  |
