# Supplementary material for: A multiresolution approach to automated classification of protein subcellular location images
Source: BMC Bioinformatics. 2007 Jun 19;8:210. doi: 10.1186/1471-2105-8-210 (PMC1933440; doi:10.1186/1471-2105-8-210)
Supplement: Additional file 1 — Compendium. 07_ChebiraBJMSMK_compendium.zip. This file is a compressed archive that contains the code that generated the results in this paper, the pseudo-code for the weighting algorithms, Table 1 with detailed results and index files of the web site containing all of this material [17]. [file 1471-2105-8-210-S1.zip › 07_ChebiraMSBJK_pseudoCode.pdf]

Algorithms

Algorithm 1 - Training Phase.

Input: d_s (local decision vector). Output: w (weight vector).

TrainingPhase(d_s)

initialize $w_{1,s}$ to classification accuracy of subband s (the percentage of correct decisions made by s over all the training images)
initialize $w_{2,s}$ to emphasize the decisions of the 0th subband
initialize $w_{3,s}$ to positive random entries
for all weight vectors, $i = 1$ to 3 **do**
  normalize w_i, initialize counter, $cnt = 0$
  compute classification accuracy with w_i and store in p_i
  while $cnt <$ maxEpochs **do**
    increment counter, $cnt = cnt + 1$
    for all images, $r = 1$ to R **do**
      set g_{dec} = the image is classified correctly with w_i
      if g_{dec} is false **then**
        for all subbands, $s = 0$ to $S - 1$ **do**
          set $l_{dec}^{(s)}$ = subband classified correctly
          if $l_{dec}^{(s)}$ is true **then**
            $w_{i,s} = w_{i,s} \cdot (1 + \epsilon)$
          else
            $w_{i,s} = w_{i,s} \cdot (1 - \epsilon)$
          end if
        end for
      end if
    end for
    compute classification accuracy with w_i and store in p_i^{new}
    if $p_i^{new} > p_i$ **then**
      set $p_i = p_i^{new}$, save w_i as w_i^{best}, reset counter, $cnt = 0$
    end if
  end while
end for
set w to w_i^{best} with the greatest p_i
return w

Algorithm 2 - Testing Phase.

Input: d_s (local decision vector), w(weight vector). Output: g (global decision vector), ACC (classification accuracy).

TestingPhase(d_s, w)

set $g = \sum_{s=1}^{S} w_s \cdot d_s$
set ACC equal to the classification accuracy of g
return g and ACC
