# Supplementary material for: A multiresolution approach to automated classification of protein subcellular location images
Source: BMC Bioinformatics. 2007 Jun 19;8:210. doi: 10.1186/1471-2105-8-210 (PMC1933440; doi:10.1186/1471-2105-8-210)
Supplement: Additional file 1 — Compendium. 07_ChebiraBJMSMK_compendium.zip. This file is a compressed archive that contains the code that generated the results in this paper, the pseudo-code for the weighting algorithms, Table 1 with detailed results and index files of the web site containing all of this material [17]. [file 1471-2105-8-210-S1.zip › 07_ChebiraMSBJK_other_Table1.pdf]

System	T	Weight.	Classification accuracy [%]						
			M (16)	T (26)	Z (49)	T, M (42)	M, Z (65)	T, Z (75)	All (91)
NMR	T_1	NW	66.12/55.37	85.49/0.57	51.20/11.55	85.76/6.71	72.48/35.53	85.06/25.60	85.04/19.58
	T_2	NW	66.12/55.37	85.76/1.84	51.20/11.55	86.64/4.26	72.48/35.53	85.78/23.49	86.24/24.57
	T_3	NW	66.12/55.37	87.46/1.79	51.20/11.55	87.38/5.83	72.48/35.53	87.12/26.70	86.86/20.45
MRB	T_3	OF	81.62/1.48	91.82/1.07	65.42/4.34	92.04/1.49	83.38/1.55	91.66/1.14	92.36/1.51
	T_3	CF	81.48/4.36	92.32/1.30	65.84/2.98	92.62/1.87	83.58/1.99	92.34/1.48	92.54/1.61
MRF	T_3	OF	84.92/0.64	94.72/0.26	65.82/2.45	94.64/0.39	86.8/0.45	94.74/0.14	94.52/0.33
	T_3	CF	85.16/1.54	95.26/0.07	65.24/3.73	95.40/0.09	85.88/1.30	92.26/0.15	95.38/0.07

Table 1: Classification accuracies and variances. Z, M and T stand for Zernicke, morphological and texture features. T_1 are the original Haralick texture features, T_2 are modified Haralick texture features and T_3 are our improved texture features. NMR denotes the base system with no MR, MRB denotes MR basis classification and MRF denotes MR frame classification. OF denotes open-form weighting algorithm while CF denotes closed-form weighting algorithm. NW denotes no weighting as there is no MR block in front. Each entry is a pair of numbers: the first one being the classification accuracy mean over a number of trials (different orderings of the images) for a given combination of feature sets; and the second one being the variance over these trials. Note that the accuracy of NMR with features M is the same across the rows T1, T2, T3 since texture features are not involved in the classification when morphological features alone are used (similarly with Z, and M, Z). A subset of these results is shown pictorially in Figure 4, to highlight the following trends: (a) Introducing MR (both MRB and MRF) significantly outperforms NMR, thus demonstrating that classifying in MR subspaces indeed improves classification accuracy. (b) MRF outperform MRB. (b) For the two versions of the weighting algorithm, open form and closed form, the closed-form algorithm slightly outperforms the open-form. (d) The trend in each case is almost flat across various feature set combinations, indicating that the texture set T_3 alone (26 features) is sufficient for high classification accuracy.
