# Supplementary figures and images for: A multiresolution approach to automated classification of protein subcellular location images
Source: BMC Bioinformatics. 2007 Jun 19;8:210. doi: 10.1186/1471-2105-8-210 (PMC1933440; doi:10.1186/1471-2105-8-210)

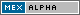

Supplement: Additional file 1 — Compendium. 07_ChebiraBJMSMK_compendium.zip. This file is a compressed archive that contains the code that generated the results in this paper, the pseudo-code for the weighting algorithms, Table 1 with detailed results and index files of the web site containing all of this material [17]. [file 1471-2105-8-210-S1.zip › 07_ChebiraMSBJK_code/lib/m2html/templates/blue/alpha.png]

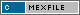

Supplement: Additional file 1 — Compendium. 07_ChebiraBJMSMK_compendium.zip. This file is a compressed archive that contains the code that generated the results in this paper, the pseudo-code for the weighting algorithms, Table 1 with detailed results and index files of the web site containing all of this material [17]. [file 1471-2105-8-210-S1.zip › 07_ChebiraMSBJK_code/lib/m2html/templates/blue/c.png]

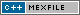

Supplement: Additional file 1 — Compendium. 07_ChebiraBJMSMK_compendium.zip. This file is a compressed archive that contains the code that generated the results in this paper, the pseudo-code for the weighting algorithms, Table 1 with detailed results and index files of the web site containing all of this material [17]. [file 1471-2105-8-210-S1.zip › 07_ChebiraMSBJK_code/lib/m2html/templates/blue/c++.png]

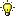

Supplement: Additional file 1 — Compendium. 07_ChebiraBJMSMK_compendium.zip. This file is a compressed archive that contains the code that generated the results in this paper, the pseudo-code for the weighting algorithms, Table 1 with detailed results and index files of the web site containing all of this material [17]. [file 1471-2105-8-210-S1.zip › 07_ChebiraMSBJK_code/lib/m2html/templates/blue/demoicon.gif]

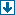

Supplement: Additional file 1 — Compendium. 07_ChebiraBJMSMK_compendium.zip. This file is a compressed archive that contains the code that generated the results in this paper, the pseudo-code for the weighting algorithms, Table 1 with detailed results and index files of the web site containing all of this material [17]. [file 1471-2105-8-210-S1.zip › 07_ChebiraMSBJK_code/lib/m2html/templates/blue/down.png]

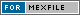

Supplement: Additional file 1 — Compendium. 07_ChebiraBJMSMK_compendium.zip. This file is a compressed archive that contains the code that generated the results in this paper, the pseudo-code for the weighting algorithms, Table 1 with detailed results and index files of the web site containing all of this material [17]. [file 1471-2105-8-210-S1.zip › 07_ChebiraMSBJK_code/lib/m2html/templates/blue/fortran.png]

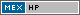

Supplement: Additional file 1 — Compendium. 07_ChebiraBJMSMK_compendium.zip. This file is a compressed archive that contains the code that generated the results in this paper, the pseudo-code for the weighting algorithms, Table 1 with detailed results and index files of the web site containing all of this material [17]. [file 1471-2105-8-210-S1.zip › 07_ChebiraMSBJK_code/lib/m2html/templates/blue/hp.png]

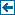

Supplement: Additional file 1 — Compendium. 07_ChebiraBJMSMK_compendium.zip. This file is a compressed archive that contains the code that generated the results in this paper, the pseudo-code for the weighting algorithms, Table 1 with detailed results and index files of the web site containing all of this material [17]. [file 1471-2105-8-210-S1.zip › 07_ChebiraMSBJK_code/lib/m2html/templates/blue/left.png]

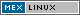

Supplement: Additional file 1 — Compendium. 07_ChebiraBJMSMK_compendium.zip. This file is a compressed archive that contains the code that generated the results in this paper, the pseudo-code for the weighting algorithms, Table 1 with detailed results and index files of the web site containing all of this material [17]. [file 1471-2105-8-210-S1.zip › 07_ChebiraMSBJK_code/lib/m2html/templates/blue/linux.png]

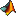

Supplement: Additional file 1 — Compendium. 07_ChebiraBJMSMK_compendium.zip. This file is a compressed archive that contains the code that generated the results in this paper, the pseudo-code for the weighting algorithms, Table 1 with detailed results and index files of the web site containing all of this material [17]. [file 1471-2105-8-210-S1.zip › 07_ChebiraMSBJK_code/lib/m2html/templates/blue/matlabicon.gif]

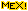

Supplement: Additional file 1 — Compendium. 07_ChebiraBJMSMK_compendium.zip. This file is a compressed archive that contains the code that generated the results in this paper, the pseudo-code for the weighting algorithms, Table 1 with detailed results and index files of the web site containing all of this material [17]. [file 1471-2105-8-210-S1.zip › 07_ChebiraMSBJK_code/lib/m2html/templates/blue/mex.png]

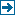

Supplement: Additional file 1 — Compendium. 07_ChebiraBJMSMK_compendium.zip. This file is a compressed archive that contains the code that generated the results in this paper, the pseudo-code for the weighting algorithms, Table 1 with detailed results and index files of the web site containing all of this material [17]. [file 1471-2105-8-210-S1.zip › 07_ChebiraMSBJK_code/lib/m2html/templates/blue/right.png]

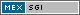

Supplement: Additional file 1 — Compendium. 07_ChebiraBJMSMK_compendium.zip. This file is a compressed archive that contains the code that generated the results in this paper, the pseudo-code for the weighting algorithms, Table 1 with detailed results and index files of the web site containing all of this material [17]. [file 1471-2105-8-210-S1.zip › 07_ChebiraMSBJK_code/lib/m2html/templates/blue/sgi.png]

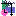

Supplement: Additional file 1 — Compendium. 07_ChebiraBJMSMK_compendium.zip. This file is a compressed archive that contains the code that generated the results in this paper, the pseudo-code for the weighting algorithms, Table 1 with detailed results and index files of the web site containing all of this material [17]. [file 1471-2105-8-210-S1.zip › 07_ChebiraMSBJK_code/lib/m2html/templates/blue/simulinkicon.gif]

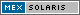

Supplement: Additional file 1 — Compendium. 07_ChebiraBJMSMK_compendium.zip. This file is a compressed archive that contains the code that generated the results in this paper, the pseudo-code for the weighting algorithms, Table 1 with detailed results and index files of the web site containing all of this material [17]. [file 1471-2105-8-210-S1.zip › 07_ChebiraMSBJK_code/lib/m2html/templates/blue/solaris.png]

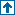

Supplement: Additional file 1 — Compendium. 07_ChebiraBJMSMK_compendium.zip. This file is a compressed archive that contains the code that generated the results in this paper, the pseudo-code for the weighting algorithms, Table 1 with detailed results and index files of the web site containing all of this material [17]. [file 1471-2105-8-210-S1.zip › 07_ChebiraMSBJK_code/lib/m2html/templates/blue/up.png]

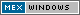

Supplement: Additional file 1 — Compendium. 07_ChebiraBJMSMK_compendium.zip. This file is a compressed archive that contains the code that generated the results in this paper, the pseudo-code for the weighting algorithms, Table 1 with detailed results and index files of the web site containing all of this material [17]. [file 1471-2105-8-210-S1.zip › 07_ChebiraMSBJK_code/lib/m2html/templates/blue/windows.png]
