# Supplementary material for: A multiresolution approach to automated classification of protein subcellular location images
Source: BMC Bioinformatics. 2007 Jun 19;8:210. doi: 10.1186/1471-2105-8-210 (PMC1933440; doi:10.1186/1471-2105-8-210)
Supplement: Additional file 1 — Compendium. 07_ChebiraBJMSMK_compendium.zip. This file is a compressed archive that contains the code that generated the results in this paper, the pseudo-code for the weighting algorithms, Table 1 with detailed results and index files of the web site containing all of this material [17]. [file 1471-2105-8-210-S1.zip › 07_ChebiraBJMSMK_compendium/index.html]

Chebira et al., A multiresolution approach to automated classificaiton
of protein subcellular location images


|  |  |  |
| --- | --- | --- |
| **<** | bimagicLab | **>** |
|  | **b****io****imag****e** **i****nformati****c****s lab** |  |

---

Jelena Kovačević
| bimagicLab

---

**Compendium**

---

Paper information and status

A. Chebira, Y.
Barbotin, C. Jackson, T.E. Merryman, G. Srinivasa, R. F. Murphy and J.
Kovačević, “A multiresolution approach to automated
classification of protein subcellular location images”, BMC
Bioinformatics, 2007.

Abstract

Background: The
problem of automated interpretation of fluorescence microscope images
depicting subcellular protein locations is at the forefront of the current
trend in biology towards understanding the role and function of all proteins.
Over the past ten years, the feasibility of using machine learning methods to
recognize all major subcellular location patterns has been convincingly
demonstrated, using diverse feature sets and combinations of
classifiers.  On a well-studied
data set of 2D HeLa single-cell images, the best performance to date, 91.5%,
was obtained upon the addition of a simple set of multiresolution features.

Results: We report
here a novel approach for the classification of subcellular location patterns
by classifying in multiresolution subspaces. Our system is able to work with
any feature set and any classifier. It consists of multiresolution (MR)
decomposition, followed by feature computation and classification in each MR
subspace, yielding local decisions that are then combined into a global
decision.  With 26 texture
features alone and a neural network classifier, we obtained an increase in
accuracy on the 2D HeLa data set to 95.3%.

Conclusions: We
demonstrate that the space-frequency localized information in the
multiresolution subspaces adds significantly to the discriminative power of
the system. Moreover, we show that a vastly reduced set of features is
sufficient, consisting of our novel modified Haralick texture features. Our
proposed system is general, allowing for any combinations of sets of features
and any combination of classifiers.

Data

2D and 3D HeLa data sets available from MurphyLab.

Code

Readme file as well as the code to generate
all the figures and tables in the paper.

[07\_ChebiraBJMSMK\_code directory]

Pseudo-code

Pseudo code for the algorithms in the paper.

[pdf]

Proofs

NA

Other material

Table 1 with variances included.

[pdf]

List of tested configurations

Matlab 7.0.1 on Linux (Rocks)

For more information or to report bugs

achebira@andrew.cmu.edu
